# Cerebral Venous Sinus Thrombosis in Iran: Cumulative Data, Shortcomings and Future Directions

**DOI:** 10.5812/ircmj.3728

**Published:** 2012-12-06

**Authors:** Afshin Borhani Haghighi, Nahid Ashjazadeh, Anahid Safari, Salvador Cruz-Flores

**Affiliations:** 1 Research Center for Stem Cell and Transgenic Technology, Shiraz University of Medical Sciences, Shiraz, IR Iran; 2 Department of Neurology, Shiraz University of Medical Sciences, Shiraz, IR Iran; 3 Research Center for Traditional Medicine and History of Medicine, Shiraz University of Medical Sciences, Shiraz, IR Iran; 4 Department of Neurology, Saint Louis University, Saint Louis, Missouri, U.S.A.

**Keywords:** Sinus Thrombosis, Intracranial, Epidemiology, Causality, Clinical Manifestations, Mortality, Iran

## Abstract

**Background:**

Cerebral venous sinus thrombosis (CVST) is a frequent cause of cerebrovascular disease in Iran.

**Objectives:**

In this study, we report cumulative data of published Iranian studies in a systematic manner with critically appraisal and presenting future directions.

**Materials and Methods:**

The authors systematically searched the ISI web of knowledge, Pubmed, Scopus, EBESCO and iranmedex for keywords attributed to cerebral venous sinus thrombosis. The methodological and demographic characteristics, etiology, site of involvement and clinical manifestations of the patients with CVST were investigated.

**Results:**

Seven eligible series with 465 patients were found. Age of the patients were between 29.5-43.8 in these series. The ratio of Female to male was 2.79. The Mortality rate was 11.4%. Oral contraceptive pills the single most common risk factor in the all series(40-71% of female patients). Headache(80-97%), sensori/motor deficits(39-64%) and seizure(20-62%) were the most common clinical presentations. Hemorrhagic transformation was seen in 11-58% of the patients. All included studies have substantial shortcomings. Majority of the studies were retrospective and only one study was population based. Despite the ethnic heterogeneity in Iran, none of these studies reported ethnic information. Detailed methodology was missing in all studies. The extent of investigation for hematologicalor neoplastic disorders was not clear methods. Only one study reported a subgroup with multifactorialetiology. Neither Barthel index nor modified Rankin scale were reported in any studies. The mortality was reported only in the three studies. The analysis of prognostic factors was not done in any study.

**Conclusions:**

To overcome theses hortcomings, more well-structured epidemiologic studies should be conducted in Iran as a CVST-raising country.

## 1. Background

Although CVST is more common in developing countries such as Iran (1), Pakistan (2), and India (3), their share in published literature is much low. For example searching "cerebral venous thrombosis" in SCOPUS search engine resulted 3808 entries (1.14.2010). Searching affiliation country "cerebral venous thrombosis" with Iran, Pakistan, India, United States of America, United Kingdom, Italy, France and the Netherlands resulted 29 (0.76%), 18 (0.47%), 135 (3.5%), 905 (23.8%), 252 (6.6%), 222 (5.8%), 356 (9.3%), 109 (2.9%), respectively.

Prevalence of CVST in Iran was reported to be12.3 per million population (1).It is higher than reports from western countries (4, 5).Mortality among Iranian patients were also higher comparing to ISCVT study (6). These necessitate well-structured studies to investigate the epidemiology, etiology, clinical and radiological manifestations, thaerapeutic options and prognosis of patients with CVST in Iran.

### 1.1. Background about Iran

Iran is a wide geographical area(1, 648, 000 km^2^) from Anatolia west to Indo-Gangetic plains of Pakistan east and from trans-Caucasus area north to Persian gulf south. Iran’s population is about about 72 million by 2008. Historically, Iran witnessed plenty of occupations and waves of migration (genetic). The major ethnic groups include Persians, Turks, Kurds, Baluchis, Arabs, and other ethinc groups. Mitochondrial DNA linage analysis showed West Eurasian as the main mtDNA linage (7).

## 2. Objectives

In this study, we reported cumulative data of published Iranian studies in a systematic manner with critically appraisal and presenting future directions.

## 3. Materials and Methods

We searched the ISI web of knowledge, Pubmed, Scopus, EBESCO and iranmedex(nation-wide search engine for Farsi articles) from 1966 to 2011. We used the following MeSH keywords as search terms: “cerebral”, “venous”, “sinus”, “ dural”, and “ thrombosis”. We also searched the reference lists of articles identified by this search strategy and selected those they judged relevant. Both Englishand Farsi studies were included in this analysis while the studies had patients in common, the case reports, reviews and Editorials were excluded the more well-structured and larger study was selected among them. We collected the all risk factors previously reported and grouped them as follows: 1) OCP and hormonal replacement, 2) pregnancy, 3) postpartum state, 4) Metabolic causes including dehydration and hyperosmotic hyperglycemia, 5) systemic inflammatory disease including systemic lupus erythematosus, rheumatoid disorders and other related conditions,and other connective tissues disorders, 6) hematological conditions including sickle cell disease and trait, coagulation disorders including hypercoagulable conditions due to activated protein C resistance, antithrombin III deficiency, factor V Leiden mutation, lupus anticoagulant, protein C deficiency, protein S deficiency, and prothrombin gene mutation, and other hematological disorders, 7) CNS infection including meningitis,encephalitis,and other CNS infections, 8) neoplastic, 9) traumatic causes, 10) miscellaneous condition not specified above, 11) idiopathic as the patient no underlying cause have been found for them.

## 4. Results

The Seven eligible studies were found. All of the reports were from teaching hospitals affliated to either Tehran (8, 9), Tabriz (10), Shiraz (11), Mashad (12), Isfahan (1, 13), and Kermanshah (14) universities of medical sciences. Tables 1, 2, 3 and 4 show methodological and demographic characteristics, ethiologies, site of involvement and clinical manifestations of patients with CVST in the found series. As we had not access to crude data calculation of age and sex , access to the specific mortality rate was also impossible.

**Table 1:** Methodological and Demographic Characteristics of Patients with CVST in Iranian Series

	City	Popultaion (2006)	Etnicity	Methodology	Date of patient recruitment	Study population	Age (Mean, range)	Female/Male	Hospital stay (Mean, days)	Follow up	Duration of f/u	Functional disability	Mortality
**Ghandehari et al (12)**	Mashhad	2,868,350	Mainly Fars	Prospective	2005-2008	62	32.3 (18 - 62)	51/11	NM	NM	NM	NM	NM
**Sahraian et al (8)**	Tehran	7,705,036	Mainly Fars	Retrospective	2003-2008	41	37.2 (15 - 75)	31/10	NM	25/41	3m	13/25	5/41 (12.2%)
**Pashapour et al (10)**	Tabriz	1,597,319	Mainly Turkish	Retrospective	2003-2006	64	43.8 (16 - 80)	55/9	NM	NM	NM	17/64 (26.6%)	5/64 (7.8%)
**Saadatnia et al (13)**	Isfahan	1,583,609	Mainly Fars	Retrospective	1997-2001	55	29.5 (17 - 71)	42/13	NM	NM	NM	NM	NM
**Janghorbani et al (1)**	Isfahan	1,583,609	Mainly Fars	Prospective	2001-2004	122	35.5 ( 17 - 70)	96/26	NM	NM	NM	NM	NM
**Ashjazadeh et al (11)**	Shiraz	1,227,331	Mainly Fars	Prospective	2000-2008	124	34.01 ± 10.25 (?-?)	87/37	NM	124/124	NM	35.48%	18/124 (14.51%)
**Shobeiri et al (14)**	Kermanshah	784,602	Mainly Kurdish	Prospective	2010	21	36.00 (18 - 55)	18/3	NM	NM	NM	NM	NM
**Salimipour et al (9)**	-	7,705,036	Mainly Fars	Retrospective	1993-1999	39	42 (14 - 19)	30/9	NM	NM	NM	NM	NM
**Total**						528		410/118 = 3.47					28/229=12.2%

**Table 2:** Predisposing Factors of Patients With CVST in Iranian Series a

	Ghnadehari (12)	Sahraian (8)	Pashapour (10)	Saadatnia (13)	Ashjazadeh (11)	Shobeiri (14)	Salimipour (9)
**Number of Patients (male/female) a**	62(51/11)	41(31/10)	64 (55/9)	55(42/13)	124(87/37)	21(18/3)	39(30/9)
**OCP and HR (% of female patients)**	29(56.8%)	22(71%)	28(50.9%)	27 (64% )	57(65.5%)	11(61.1%)	12(40%)
**Pregnancy (% of female patients)**	3(5.9%)	3(9.7%)	-	3(7.1%)	10(11.5%)	1(5.5%)	-
**Post partum (% of female patients)**	2(3.9%)	NM	9(16.4%)	4(9.5%)	10(11.5%)	NM	1(3.3%)
**Dehydration (metabolic diseases)**	18(29%)	NM	NM	NM	NM	NM	NM
**Inflammatory diseases b**	1(1.6%)	NM	2(3.1%)	6(10.9%)	13(10.5%)	NM	NM
**Hematologic**	NM	NM	NM	2(3.6%)	NM	NM	NM
**Hypercoagulable state c (included APL)**	9(13.8%)	NM	15(23.4%)	9(16.4%)	5 (4%)	1(4.8%)	1(2.6%)
**Infection**	1(1.6%)	3(7.3%)	2(3.1%)	4(7.2%)	11(8.9%)	NM	9(30%)
**Trauma**	NM	2(4.9%)	NM	2(3.6%)	NM	NM	NM
**Neoplasm**	1(1.6%)	3(7.3%)	NM	2(3.6%)	NM	NM	NM
**Miscellaneous **	2(3.2%)	NM	NM	NM	12(9.7%) d	NM	NM
**Idiopathic**	15(24.2%)	NM	8(12.5%)	11(20%)	16(12.9%)	NM	8(20.5%)

^a^In all series some patients had multiple contributing factors

^b^Inflammatory Diseases: Inflammatory connect tissue diseases2011 ICD-9-CM Diagnosis Code 710 and 714 and inflammatory bowel disease

^c^Hypercoagulable state: included in 2011 ICD-9-CM Diagnosis Code 289.81 and 289.82

^d^Miscelalnous includes malignancy, trauma and liver diseases

**Table 3:** Site Involvement in Patients With CVST in Iranian Series (Any Involvement Single or Combined )

	Ghandehari (12)	Sahrarian (8)	Pashapour (10)	Saadatnia (13)	Ashjazadeh (11)	Shobeiri (14)	Salimipour (9)
**Patients, No.**	62	41	64	55	124	21	39
**Superior sagital**	NM	11(26.8%)	50(78.1%)	40(72%)	80%	15(71.4%)	18(46.2%)
**Transverse (lateral)**	NM	6(14.6%)	13(20.3%)	11(20%)	46%	20(95.2%)	18(46.2%)
**Sigmoid**	NM	3(7.3%)	6(9.4%)	NM	NM	11(52.4%)	NM
**Staright**	NM		NM	NM	NM	2(9.5%)	NM
**Cavernous**	NM	1(2.4%)	NM	NM	5.2%	NM	2(5.1%)
**Deep vein**	NM	NM	NM	3(5.4%)	3.2%	NM	NM
**Cortical vein**	NM	NM	NM	NM	NM	4(19%)	NM
**Jugular vein**	NM	NM	NM	1(1.8%)	NM	3(14.3%)	NM

**Table 4:** Clinical Manifestations of Patients With CVST in Iranian Series

	Ghandehari (12)	Sahraian (8)	Pashapour (10)	Saadatnia (13)	Ashjazdaeh (11)	Shobeiri (14)	Salimipour (9)
**Headache**	NM	33 (80%)	62 (96.9%)	52 (94.5%)	116 (93.54%)	20 (95.2%)	NM
**Papilledema**	NM	4 (9.75%)	45 (70.3%)	40 (72.7%)	48 (62.3%)	NM	NM
**Mental disorder a**	NM	8 (19.5%)	2 (3.12%)	25 (45.4%)	31 (25%)	NM	NM
**Sensorimotor Deficit b**	NM	16(39%)	41 (64%)	25 (45.4%)	44 (35.48%)	NM	NM
**Seizure**	NM	10 (20.4%)	25 (39%)	32 (58.1%)	28 (36.4%)	10 (47.62%)	NM
**Hemorrhage c**	NM	24 (58.5%)	15 (23.4%)	6 (10.9%)	NM	10 (47.6%)	NM

^a^Acute confusional state, delirium, stupor, coma, Mental disturbances

^b^Unilateral senory and/or motor changes

^c^Parenchymal or subarachnoid hemorrhage

Mean delay in diagnosis was reported as 16.2 days in one report (8). The Length of Stay Hospitalization which was only reported in the same series was 14.8 days (4-42) (8). A local problem, hardly if ever seen in western countries is OCP use due to religious cause. As per Islamic rules Ramadan fasting should not be done during menstruation. In the mean time,the women must compensate for the broken days of obligatory fasting. Some rituals of hajj pilgrimage should also not to be conducted by menstruating women. Some women who wants to be able to fast or do hajj pilgrimage along with the other Muslims, obtain OCPs to delay menstruation during Ramadan month or haj event (15).

19 out of 62 patients in Ghandehari et al, (12) and 6 out of 20 patients in Sahraian et al (8) series who developed CVST due to OCP, took the medication for prevention of menstruation to be able to do religious rituals like Ramadan dieting or Hajj pilgrims. Same phenomenon has been reported in the other parts of Iran (6, 15, 16). Dehydration in Ramadan dieting and immobilization in long -journey for Hajj pilgrim may also be contributing factors.

## 5. Discussion

All included studies have substantial shortcomings. As it is shown in tables, there were lots of unmentioned measures in each study. Majority of the studies were retrospective (8-10, 14). Only one study was population based (13). Estimation of the incidence and prevalence metrics from other studies were impossible. Demographic information of patients was not thoroughly reported. Despite ethnic heterogeneity in Iran, none of these studies reported ethnic information of the patients populations. Pashapoor et al (10) study and Shobeiri et al (14) study was done in cities with majority of Turkish and Kurdish populations, respectively. But ethnicity of the patients was not mentioned specificly in these studies. origin of the patients were not mentioned either. The Length of hospitalization was only reported in one series (8). Medicoeconomic parameters such as type of insurance coverage, total hospital charge and disposition of discharge has not been reported in none of the studies.

The inclusion and exclusion criteria were not thoroughly defined in these studies. The detailed laboratory investigations were not mentioned as well. For example the extent of hematological investigation for thrombophilic states or oncological studies in neoplastic disorders were not mentioned in methods part.

Multiple risk factors can be contributory in ethiopathologenesis of CVST. Therefore, detection of one risk factor should not deter researchers from investigation for other causes (17). Only one study reported a subgroup with multifactorial ethiologies (12). National Institute of Health Stroke Scale (NIHSS), Barthel index, or much more simply, modified Rankin scale (mRS) as quantitative measures of disabilities at time of discharge were not reported in any studies. Only one study has midterm follow-up (median 3 months) in majority of patients (8). Even in this study, the functional disability was not reported with mRS.

Mortality was reported only in the three studies (8, 10, 11). As a statistical drawback, multivariate analysis of poor prognostic factors was not done in any study. The selection bias was also presented,in a center with interest to neurological manifestations of Behcet’s disease with active screening of neurological manifestation of Behcet’s disease (18),8.2% of patients had Behcet’s disease. In another report that radiologists are more active, the frequency of involved sinuses are dramatically different from other studies.

The current Iranian studies do not address unanswered questions including interaction of different etiologies, predilection of some(but not all) women who use OCP to CVST, poor prognostic factors which necessitate supplementary therapeutic options like thrombolysis or thrombectomy, medicoeconomic burden of CVST, etc. Iran centers should upgrade their logistics and overcome above-mentioned methodological drawbacks to join international studies for CVST. National Practice guidelines for CVST should be written too.

## References

[1] Janghorbani M, Zare M, Saadatnia M, Mousavi SA, Mojarrad M, Asgari E (2008). Cerebral vein and dural sinus thrombosis in adults in Isfahan, Iran: frequency and seasonal variation.. Acta Neurol Scand..

[2] Khealani BA, Wasay M, Saadah M, Sultana E, Mustafa S, Khan FS (2008). Cerebral venous thrombosis: a descriptive multicenter study of patients in Pakistan and Middle East.. Stroke..

[3] Kalita J, Bansal V, Misra UK, Phadke RV (2006). Cerebral venous sinus thrombosis in a tertiary care setting in India.. QJM..

[4] Stam J (2003). Cerebral venous and sinus thrombosis: incidence and causes.. Adv Neurol..

[5] Ferro JM, Correia M, Pontes C, Baptista MV, Pita F (2001). Cerebral vein and dural sinus thrombosis in Portugal: 1980-1998.. Cerebrovasc Dis..

[6] Azin H, Ashjazadeh N (2008). Cerebral venous sinus thrombosis--clinical features, predisposing and prognostic factors.. Acta Neurol Taiwan..

[7] Quintana-Murci L, Chaix R, Wells RS, Behar DM, Sayar H, Scozzari R (2004). Where west meets east: the complex mtDNA landscape of the southwest and Central Asian corridor.. Am J Hum Genet..

[8] Sahraian MA, Akbari H, Khajavi MR, Najafi A, Khashayar P (2010). The risk factors and the treatment course of cerebral venous thrombosis: an experience of 41 cases.. Acta Neurol Belg..

[9] Salimipour H, Nafisi S, Sikaroudi H, Lotfi J (2001). Cerebrospinal Fluid Analysis in Cerebral Venous Sinus Thrmbosis.. Teb Jonub..

[10] Pashapoor A, Arami M, Valaee A (2007). Cerebral venous thrombosis in adults: a clinical study of 64 Iranian cases.. Internet J Neurol..

[11] Ashjazadeh N, Borhani Haghighi A, Poursadeghfard M, Azin H (2011). Cerebral Venous-Sinus Thrombosis: A Case Series Analysis.. Iran J Med Sci..

[12] Ghandehari K, Shams M, Atalu A, Afzalnia A, Ahmadi F, Khazaei M (2009). Oral contraceptive consumption and cerebral venous thrombosis in Mashhad, Iran.. Internet J Neurol..

[13] Saadatnia M, Mousavi SA, Haghighi S, Aminorroaya A (2004). Cerebral vein and sinus thrombosis in Isfahan-Iran: a changing profile.. Can J Neurol Sci..

[14] Shobairi E, Razazian N, Rezaie M, Esmaeli MRS (2010). Incidence rate of cerebral venous thrombosis and its related factors in Kermanshah city in 2009-2010.. Sci J Kurdistan Univ Med Sci..

[15] Saadatnia M, Zare M, Fatehi F, Ahmadi A (2009). The effect of fasting on cerebral venous and dural sinus thrombosis.. Neurol Res..

[16] Saidee M, Froghipoor M, Sasannejad P, Mellat Ardakani A, Azarpazhooh M (2008). The relation between short course oral contraceptive consumption and cerebral vein thrombosis in Ramadan.. Iran J Neurol..

[17] Ferro JM, Canhao P, Stam J, Bousser MG, Barinagarrementeria F (2004). Prognosis of cerebral vein and dural sinus thrombosis: results of the International Study on Cerebral Vein and Dural Sinus Thrombosis (ISCVT).. Stroke..

[18] Ashjazadeh N, Borhani Haghighi A, Samangooie S, Moosavi H (2003). Neuro-Behcet's disease: a masquerader of multiple sclerosis. A prospective study of neurologic manifestations of Behcet's disease in 96 Iranian patients.. Exp Mol Pathol..

